# Identification of Bacteriology and Risk Factor Analysis of Asymptomatic Bacterial Colonization in Pacemaker Replacement Patients

**DOI:** 10.1371/journal.pone.0119232

**Published:** 2015-03-13

**Authors:** Xian-Ming Chu, Hua Yu, Xue-Xia Sun, Yi An, Bing Li, Xue-Bin Li

**Affiliations:** 1 Department of Cardiology, the Affiliated Hospital of Qingdao University, Qingdao, 266100, China; 2 Department of Biology, Medical College of Qingdao University, Qingdao, 266021, China; 3 The Affiliated Cardiovascular Hospital of Qingdao University, Qingdao, 266000, China; 4 Department of Cardiac Electrophysiology, Peking University People's Hospital, Beijing, 100044, China; University Hospital San Giovanni Battista di Torino, ITALY

## Abstract

**Background:**

Recent researches revealed that asymptomatic bacterial colonization on PMs might be ubiquitous and increase the risk of clinical PM infection. Early diagnosis of patients with asymptomatic bacterial colonization could provide opportunity for targeted preventive measures.

**Objective:**

The present study explores the incidence of bacterial colonization of generator pockets in pacemaker replacement patients without signs of infection, and to analyze risk factors for asymptomatic bacterial colonization.

**Methods:**

From June 2011 to December 2013, 118 patients underwent pacemaker replacement or upgrade. Identification of bacteria was carried out by bacterial culture and 16S rRNA sequencing. Clinical risk characteristics were analyzed.

**Results:**

The total bacterial positive rate was 37.3% (44 cases), and the coagulase-negative *Staphylococcus aureus* detection rate was the highest. Twenty two (18.6%) patients had positive bacterial culture results, of which 50% had coagulase-negative *staphylococcus*. The bacterial DNA detection rate was 36.4 % (43 cases). Positive bacterial DNA results from pocket tissues and the surface of the devices were 22.0% and 29.7%, respectively. During follow-up (median, 27.0 months), three patients (6.8%, 3/44) became symptomatic with the same genus of microorganism, *S*. *aureus* (n=2) and *S*. *epidermidis* (n=1). Multivariable logistic regression analysis showed that history of bacterial infection, use of antibiotics, application of antiplatelet drugs, replacement frequency were independent risk factors for asymptomatic bacterial colonization.

**Conclusion:**

There was a high incidence of asymptomatic bacterial colonization in pacemaker patients with independent risk factors. Bacterial culture combined genetic testing could improve the detection rate.

## Introduction

Cardiovascular implantable electronic devices (CIEDs) have saved countless lives since 1950s [1]. However, consequent PM-related infection has become a clinical problem that is difficult to treat and associated with high fatality rate. It has been reported that PM infection rate was 1~7% [2–4]. So, it is very necessary to explore risk factors for PM infection, which could provide basis for targeted preventive measures.

Bacterial biofilms and bacterial colonization on the surface of *in vivo* implanted devices might lead to clinical infection[5–8]. Recent researches revealed that asymptomatic bacterial colonization on PMs might be ubiquitous and increase the risk of clinical PM infection[9–12]. Early diagnosis of patients with asymptomatic bacterial colonization is an important basis for applying specific preventive measures and reducing clinical PM infection. In the present study, both traditional culture and 16S rRNA gene sequencing were carried out to identify the bacteria in pocket tissues and on the surface of generators in patients with replacement of PMs. We also analyzed the related risk factors for bacterial colonization and clinical PM infection.

## Methods

### 2.1. Patients and procedure

Between June 2011 and December 2013, a total of 118 patients who had replaced or upgraded pacemakers were included in this study. Patients were excluded if they were clinically diagnosed with PM infection, including pocket infection, bacteremia, and infective endocarditis. Clinical characteristics and laboratory examination results were collected, and prospective follow-up were carried out. Based on the Declaration of Helsinki, all patients signed medical informed consent forms to participate in this study, and the study was approved by the ethics committee of the Affiliated Hospital of Qingdao University.

Routine checks included a chest X-ray and a cardiac color ultrasound. Before the operation, routine blood tests were carried out. The first generation of cephalosporin antibiotics was injected once pre-operation and persisted for 72 hours after the operation. Patients were subjected to a chest X-ray, wound check, and routine pacemaker program control follow-up before leaving the hospital one week after the operation. Routine follow-up was carried out for all patients every three months after the operation. Clinical symptoms included local inflammation in the pocket tissue, including erythema, fever, fluctuation, wound dehiscence, decay, tenderness, and suppuration. The diagnosis of infective endocarditis was according to the European Society of Cardiology (ESC) criteria [13].

### 2.2. Collection of clinical characteristics

The clinical characteristics, medical history, comorbidities and laboratory examinations were collected. Bacterial infection history in the past five years contained upper respiratory infection, lower respiratory infection, urinary system infection, soft tissue infection, digestive system infection, and infection in other parts. History of surgery referred to recorded surgery required hospitalization in the past five years. The comorbidities definitions: renal insufficiency (glomerular filtration rate < 60ml/min×1.72m^-2^), systolic heart failure (NYHA ≥ II class, ejection fraction < 45%), and chronic heart disease (diagnosed coronary heart disease, NYHA classes III and IV, or hypertension that needed to be treated by ≥3 drugs). Antibiotic therapy was defined as any sequential oral or intravenous antibiotic therapy for more than seven days in the past five years.

### 2.3. Specimens

Before generator was removed, pocket tissue was sampled and biofilms on the surface of the generators were collected using a sterile scalpel. Before the operation, all surgical instruments were soaked in the sterile saline for 20 minutes, and then the soaking liquid was cultured. The venous blood of all patients was collected for culture 24 hours before and after operation.

### 2.4. Microbiological culture

Specimens were collected in sterile specimens boxes and sent to microbiology laboratory within 30 minutes. Microbiological experiments began in 6 hours. The tissues were grinded under the aseptic conditions, and then inoculated in blood agar medium, China blue medium and chocolate medium, and at the same time the broth was inoculated to increase bacteria. All medium and broth were cultured in 35°C incubator. Bacterial growth was observed 24 hours later.

### 2.5. 16S rRNA genetic testing [14]

Pocket tissues and the samples obtained from the surface of generators were washed with phosphate buffer solution (PBS). Genomic DNA was extracted using Wizard genomic DNA extraction kit according to the manufacturer’s protocol.

In order to accurately determine the bacteria in the sample, universal primers (upstream primer: AGAGTTTGATCCTGGCTCAG; downstream primer: AGTAAGGAGGTGATCCAACCGCA) were designed to target the conserved region of the 16S rRNA gene (rDNA) according to *Escherichia coli* sequence, which could amplify nearly all bacteria. The positive band indicated the presence of bacteria in the sample. The PCR product was purified using Wizard PCR Preps DNA Purification System and then ligated into the pGEM-T Easy Vector. The ligation product was transformed into the *E*. *coli* strain JM109. Colonies containing 16S rRNA genes were identified using blue/white screening. Plasmid DNA from candidate colonies was extracted and digested with *Eco*RI. The inserted 16S rRNA gene was then sequenced and identified using the BLAST algorithm against EMBL and GenBank databases.

### 2.6. Statistical analysis

Normally distributed continuous variables were expressed as means±SD, and continuous variables of skewed distribution were expressed as median values. Comparison between two continuous variables of normal distribution was carried out using t-test. Comparison of classified variables was performed using Chi-square test or Fisher's exact test. Correlation between clinical characteristics and PM asymptomatic bacterial colonization was analyzed using multivariate logistic regression analysis.

## Results

### 3.1. Microbiological results

The total bacterial positive rate was 37.3% (44 cases) (Table 1),and the coagulase-negative Staphylococcus aureus detection rate was the highest (Table 2 and 3). Positive culture rate of pocket tissues were 18.6% (22 patients), of which coagulase-negative staphylococcus accounted for 50.0% (11 cases) (Table 1 and 2). The culture of venous blood and instruments soaking solution were all negative.

**Table 1:** Bacterial results of 118 patients.

methods	Positive patients (n)	Percentage (%)
**Overall**	44	37.3
**Bacterial culture**	22	18.6
**16S rDNA**	43	36.4
**Both methods**	21	17.8

**Table 2:** Bacterial culture results of pocket tissues (n = 22, 18.6%).

Species	Positive patients (n)	Percentage (%)
***Staphylococcus aureus***	2	9.1
***Coagulase-negative Staphylococcus***	11	50
*** S*. *epidermis***	6	27.3
*** S*. *warneri***	4	18.2
*** S*. *hominis***	1	4.5
***Streptococcus viridans***	**2**	9.1
***Pseudomonas aeruginosa***	**1**	4.5
***Propionibacterium acnes***	**1**	4.5
***Klebsiella pneumoniae***	**1**	4.5
***Enterobacter cloacae***	**1**	4.5
***Escherichia coli***	**1**	4.5
***Acihetobacter baumanii***	**1**	4.5
***Serratia marcescens***	**1**	4.5

**Table 3:** Bacterial species determined by DNA technology.

Species	Positive specimens (n = 61)	Percentage (%)
***Staphylococcus aureus***	**3**	**4.9**
***Coagulase-negative Staphylococcus***	**19**	**31.1**
*** S*. *epidermis***	**8**	**13.1**
*** S*. *saprophyticus***	**3**	**4.9**
*** S*. *warneri***	**5**	**8.2**
*** S*. *hominis***	**3**	**4.9**
***Streptococcus viridans***	**4**	**6.6**
***Pseudomonas aeruginosa***	**4**	**6.6**
***Propionibacterium acnes***	**4**	**6.6**
***Corynebacterium parvum***	**4**	**6.6**
***Klebsiella pneumoniae***	**4**	**6.6**
***Enterobacter cloacae***	**5**	**8.2**
***Escherichia coli***	**6**	**9.8**
***Acihetobacter baumanii***	**3**	**4.9**
***Stenotrophomonas maltophilia***	**2**	**3.3**
***Serratia marcescens***	**1**	**1.6**
***Enterococcus faecalis***	**1**	**1.6**
***Granulicatella adiacens***	**1**	**1.6**

**Fig 1:**
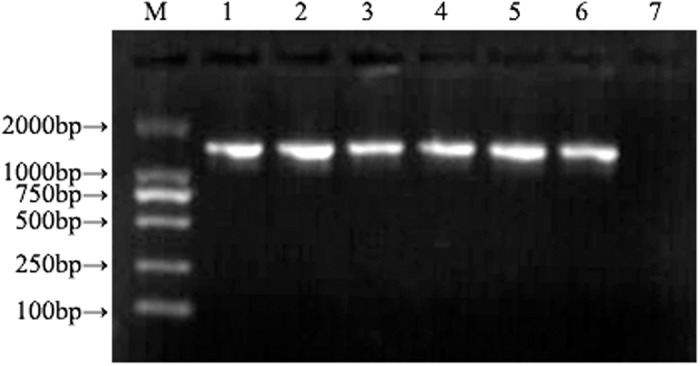
UP-PCR results of some patients. We have detected all the patients and finally we chose some positive bacteria for the electrophoresis analysis. The deeper the band, the more the bacteria. 1–6 represented PCR product of the 16S rRNA gene of *Staphylococcus epidermidis*, *Pseudomonas aeruginosa*, *Escherichia coli*, *Streptococcus viridans*, *Corynebacterium parvum*, and *Klebsiella pneumoniae*; M represented DL2000 DNA Marker; 7 represented negative control.

**Fig 2:**
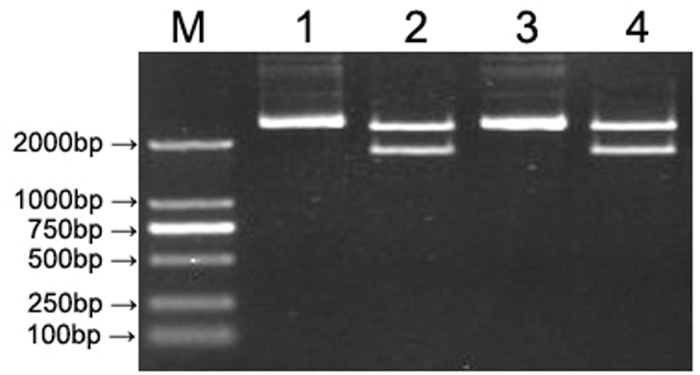
Identification of enzyme digestion of 16S rRNA gene recombinant. 1, 3 represented blank pGEM-T plasmid; 2, 4 represented enzyme-digested product of 16S rRNA gene recombinant plasmid; M represented DL2000 DNA Marker.

**Fig 3:**
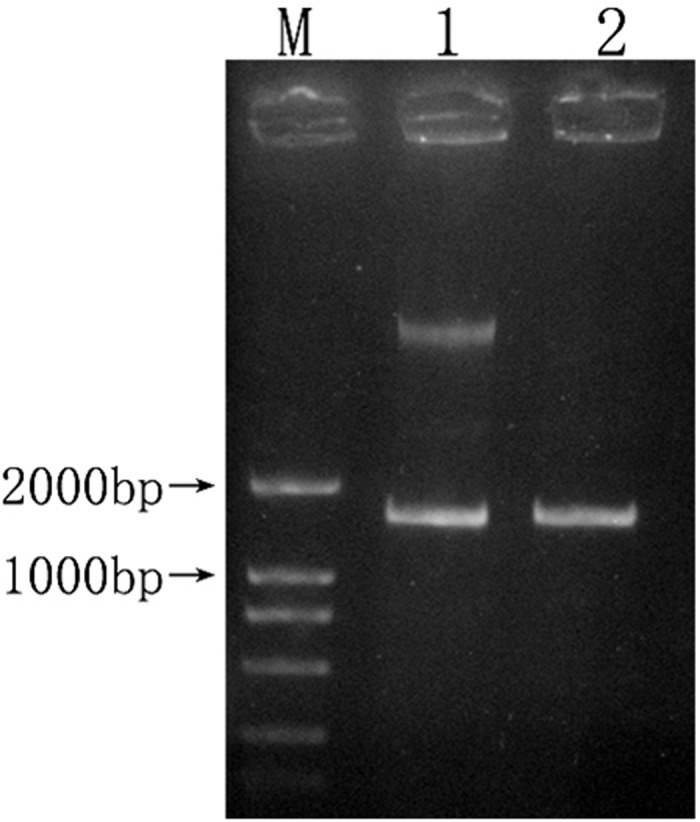
Enzyme digestion result of 16S rRNA gene recombinant. 1 represented enzyme-digested product of 16S rRNA gene recombinant plasmid; 2 represented PCR product of 16S rRNA gene;M represented DL2000 DNA Marker.

PCR amplification results of the 16S rRNA gene from partial patients are shown in Fig. 1. The amplified fragment length was 1532bp. Identification results of restriction enzyme digestion of recombinant plasmid are shown in Fig. 2 and Fig. 3. At least three clones from each PCR ligation reaction per patient were sequenced for the possibility of coexistence of diverse bacteria.

Identification results of the bacterial 16S rRNA gene are shown in Table 1, 3 and 4. Bacteria were detected in 36.4% of 118 patients, among which 22.0% were found in pocket tissues and 29.7% in biofilms (the samples obtained from the surface of generators). The percentage of coagulase-negative *Staphylococcus* was the highest (31.1%).

**Table 4:** DNA results of 118 patients.

Specimen	Positive patients (n)	Percentage (%)
**Overall**	43	36.4
**Pocket tissue**	26	22.0
**Surface of device**	35	29.7
**Both specimens**	18	15.3

### 3.2. Related risk factors

Single factor analysis and multivariate logistic regression analysis of asymptomatic bacterial colonization risk factors are shown in Tables 5–8.

**Table 5:** Bacterial detection results of 118 patients.

	positive(n = 44)	negative(n = 74)	*χ* ^*2*^	P
**Age**	69.1±15.2	67.8±17.1	0.4158	0.6783
**Gender**				
** Male**	25	37	0.514	0.473
** Female**	19	37		
**PM indications**				
** SSS**	20	31	0.790	0.674
** AVB**	15	31		
** AF with long intervals**	9	12		
**Replacement time**				
** 1 time**	30	68		0.003^a^
** 2 times**	11	5		
** 3 times**	3	1		
**PM types**				0.829^a^
** Single chamber**	15	24		
** Double chamber**	27	48		
**ICD/CRT**	2	2		

PM, pacemaker; SSS, sick sinus syndrome; AVB, atrioventricular block; AF, atrial fibrillation; ICD, implantable cardioverter-defibrillator; CRT, cardiac resynchronization therapy.

^a^: Fisher Exact Test

**Table 6:** Physiological characteristics of 118 patients.

	bacteria positive(n = 44)	bacteria negative(n = 74)	t	P
**Body mass index**	25.26±4.74	25.96±4.01	0.8561	0.3937
**White blood cells (×10** ^**9**^ **/L)**	6.25±2.1	6.07±2.3	0.4244	0.6721
**Blood platelets (×10** ^**9**^ **/L)**	230.64±39.24	227.76±35.64	0.4087	0.6835
**Hemoglobin (g/L)**	136.96±8.67	139.59±8.12	1.6589	0.0998
**Total serum protein (g/L)**	63.72±6.48	64.68±7.03	0.7382	0.4619
**Serum albumin (g/L)**	41.88±4.14	43.92±4.20	2.5649	0.0116
**Ejection fraction (EF %)**	53.67±7.88	56.56±7.28	2.0220	0.0455

**Table 7:** Comorbidities and drug application.

	bacteria positive(n = 44)	bacteria negative(n = 74)	*χ* ^*2*^	P
**Coronary heart disease**	12	20	0.001	0.977
**Hypertension**	22	32	0.508	0.476
**Atrial fibrillation**				
** Paroxymal**	6	7	0.089	0.957
** Persistent**	4	6		
** Permanent**	6	8		
**Dilated cardiomyopathy**	8	9	0.811	0.368
**Diabetes**	10	12	0.771	0.380
**Renal insufficiency**	10	5	6.343	0.012
**Chronic systolic HF**	10	16	0.020	0.889
**COPD**	4	4	0.593	0.441
**Immunosuppressor**	1	2		1.000^a^
**Warfarin**	3	3	0.052	0.820
**Antiplatelet drug use**	24	18	10.993	0.001
**Antibiotic use**	12	6	7.840	0.005
**Malignancy**	1	2		1.000^a^
**Bacterial infection history**	34	16	34.997	<0.0001
**Surgical history**	6	7	0.157	0.692

HF, heart failure; COPD, chronic obstructive pulmonary disease.

^a^: Fisher Exact Test

**Table 8:** Multi-factor logistic regression analysis.

Risk factor	β	SE (β)	Wald	P	OR	95%CI
**Bacterial infection history**	**2.684**	**0.832**	**18.74**	**<0.0001**	**14.644**	**2.867–74.793**
**Antibiotic use**	**1.568**	**0.684**	**8.342**	**0.003**	**4.797**	**1.255–18.332**
**Antiplatelet drug use**	**1.336**	**0.456**	**6.457**	**0.006**	**3.804**	**1.556–9.298**
**Replacement of two times**	**1.712**	**0.820**	**4.017**	**0.033**	**5.540**	**1.110–27.638**

Replacement times: significant difference between 2 and 1 times, no difference between 3 and 1 times.

### 3.3. Clinical follow-up

The median follow-up time was 27.0 months (range, 9–39 months; mean 25.9 months). During the follow-up, two death occurred because of myocardial infarction and heart failure, respectively. PM infection occurred 3, 7, and 11 months after operation respectively in three (6.8%) patients, and the pathogenic bacteria were *S*. *aureus* (n = 2) and *S*. *epidermidis* (n = 1), which were consistent with bacteria identified in replacement. One patient had cancer, and infective endocarditis was confirmed by wire vegetations and positive blood culture of *S*. *aureus*. The other two patients showed pocket infection.

## Discussion

PM infection has became serious complication in clinical practice[2–4, 15–16]. The bacteria leading to apparent pocket infection or lead infection can be cultured and identified. However, as a potential infection, only 20–30% of bacteria could be identified by traditional culture-dependent methods. It is not well understood whether the pathogens are not culturable or if it is aseptic inflammation [17–20]. How many patients carry bacteria but show no symptoms? What bacteria are present? What is the relationship between the bacteria and clinical infection? All these questions remain unanswered.

In addition to bacterial culture, the 16S rRNA gene sequence analysis technology has revolutionized bacterial taxonomy. The homology of ancient 16S rRNA is high, and the gene contains both conserved and variable sequences. The molecular size of the gene is suitable to operate and the sequence variation adapts to evolutionary distance. Therefore, the 16S rRNA gene has become important and useful method in bacterial taxonomy [20, 21]. Provided that bacterial DNA was rapidly degraded after death [21, 22], it could be considered that the organism detected was derived from micro functional groups but not molecular residues from contamination during the last operation.

In the present study, more than a third (37.3%) of the patients had positive bacteria. The major bacterium was coagulase-negative *Staphylococcus*. The high positive results were in line with previous studies [9, 12, 23]. Compared with the relative lower culture positive rate (18.6%), DNA was isolated from 29.7% of biofilms on the generator surface and 22.0% of subcutaneous pocket tissues, and 15.3% from both. This result showed that microbes were easy to exist not only on the generator surface but also in tissues near the generator. The bacterium that was considered to grow on the skin, e.g. *C*. *parvum*, may be caused by contamination.

During the follow-up period, three patients (6.8%) suffered from clinical infection with the common pathogenic bacteria of CIED infection in the previous study [16]. While the vast majority of patients with positive bacteria had no clinical symptoms of infection. This prompted that there were microbial communities around the generators, and some conditional pathogenic bacteria (e.g. *Staphylococcus*) might lead to infection. What conditions can lead to clinical infection is the key to the problem. There were hypotheses which could explain an equilibrium between the human host and bacteria colonization [24]. When the balance is broken, bacteria are destroyed or infection occurs. Many factors may influence this balance, such as the number of bacteria or the addition of a new infection, the virulence of bacteria and their ability to adapt to the unfavourable environment, and the defensive capacity of the host[9–12].

There are factors associated with a weakened immune response or predisposition to repetitive bacteraemias, which have been shown to predispose to the infection[25]. The risk factors include renal failure[19, 26], diabetes and congestive heart failure [21], the number of previous operations[20, 27], increasing number of leads [22], experienced bacteraemias [28], and vegetations on the leads [25, 29]. But a previous study showed that common risk factors for device infection did not correlate with the presence of DNA [9].

In the present study, analyses of risk factors related to asymptomatic bacterial colonization indicated that bacterial infection history, antibiotic history, usage of antiplatelet drugs and twice PM replacement were independent risk factors for asymptomatic bacterial colonization. The history of bacterial infection and antibiotic usage prompted immune dysfunction, and the patients might be repeatedly exposed to bacterial infection or bacteremia, which might increase the pathogenic bacteria migration to the surface of implants in the body. Use of antiplatelet drugs may cause micro bleeding in pockets, and renal insufficiency was often associated with immune and circulation dysfunction, which might be susceptible to microbial colonization.

Repeating interventional treatments decrease patients’ defense to pathogens and increase the probability of infection [2–4, 15]. Research indicated that one-third of implantable cardioverter-defibrillator (ICD) patients were positive for microbial swab culture in pocket tissues and drawn wires when replacing generator and wires [10]. The probability of infecting complications significantly increased for the patients who received treatment for a complex implantable device [30, 31]. The infection rate was 5.5% for the young patients that received an average of two pacemaker implanting operations, five times higher than that for the first received [30]. Secondary intervention for hematomas and movement of the wire were the two factors that easily lead to infection, and the odds ratio (OR) reached up to 15.04 [30]. Harcombe et al. revealed that the occurrence rate of complications caused by PM replacement was 6.5%, while that caused by first PM implantation was 1.4% [32]. The complications resulted from the erosion and infection of the implantable device [32]. In total, 80% of patients with clinical infection had received on average twice or more than twice the PM implanting operation, suggesting that repeated implanting significantly increased the probability of infection. The infection probability of first implantation was 0.8% and 4% for replacement of the device [3, 32].

There were several hypotheses for the source and mechanism of CIED infection. One was contamination of the pocket tissue. After implantation, patients’ defense capabilities shifted and previously dormant bacteria massively propagated, leading to infection [24]. Under this hypothesis, asymptomatic bacterial colonization led to infection after the device-replacing operation, which explained why the infection rate of the second implantation was higher than that of the first. Bacterial contamination was related to the operating time and became a tough problem with more and more patients receiving ICD and/or cardiac resynchronization therapy (CRT). The ICD patients who suffered serious heart trouble were easier to be infected, which would affect the survival time [33]. In addition, remnants of CIED *in vivo* may increase the risk of infection. Complete removal of all CIED hardware should be attempted at the time of upgrade, revision, and even prior to orthotopic heart transplantation [34, 35]. Furthermore, gene polymorphisms, such as fibronectin-binding protein A of *S*. *aureus*, are associated with infection of cardiovascular devices [36].

## Limitations

Although traditional culture combined 16S rRNA gene sequencing were carried out to improve detection sensitivity, the possibility of false negative and positive results must be considered. Interference factors contained the features of PMs, antibiotic therapeutic regimen, the location of the implanting pocket, laboratory conditions, and contamination. Although quantitative PCR was used, it was impossible to quantify the bacteria completely on and around the PM, which might be an important factor for pathogenicity. The joint of more advanced technologies may be a solution. Recent research has indicated that means of sonic degradation was conducive to bacterial determination after replacement, removal, and infection [37–39]. In addition, the follow-up time was relatively short. With the extension of time, there may be more clinical infection, and then, some important issues could be answered, such as whether asymptomatic bacterial colonization could lead to clinical infection.

## Conclusions

The asymptomatic bacterial colonization in PM patients was widespread with independent risk factors. Some patients with bacteria related to common microflora in PM infection became clinical infection. However, the exact mechanisms and functios of these bacteria need further research, such as protective, conditional pathogenic, or collaborative. In order to kill bacteria in particular organizations, new treatment methods were being constantly generated. A recent study revealed that direct current can kill *S*. *aureus* in biofilms on the surface of PMs [40].
